# Ferroptosis and iron metabolism in diabetes: Pathogenesis, associated complications, and therapeutic implications

**DOI:** 10.3389/fendo.2024.1447148

**Published:** 2024-08-30

**Authors:** Eun-Ju Jin, Yunju Jo, Shibo Wei, Manfredi Rizzo, Dongryeol Ryu, Karim Gariani

**Affiliations:** ^1^ Department of Biomedical Science and Engineering, Gwangju Institute of Science and Technology, Gwangju, Republic of Korea; ^2^ College of Medicine, Mohammed Bin Rashid University of Medicine and Health Sciences, Dubai, United Arab Emirates; ^3^ Department of Health Promotion Sciences, Maternal and Infant Care, Internal Medicine and Medical Specialties (PROMISE), University of Palermo, Palermo, Italy; ^4^ Service of Endocrinology, Diabetes, Nutrition, and Therapeutic Education, Faculty of Medicine, Geneva University Hospitals, Geneva, Switzerland; ^5^ Diabetes Center of the Faculty of Medicine, University of Geneva Medical School, Geneva, Switzerland

**Keywords:** ferroptosis, diabetes, iron, treatment, complications

## Abstract

Diabetes mellitus is a complex chronic disease, considered as one of the most common metabolic disorders worldwide, posing a major threat to global public health. Ferroptosis emerges as a novel mechanism of programmed cell death, distinct from apoptosis, necrosis, and autophagy, driven by iron-dependent lipid peroxidation accumulation and GPx4 downregulation. A mounting body of evidence highlights the interconnection between iron metabolism, ferroptosis, and diabetes pathogenesis, encompassing complications like diabetic nephropathy, cardiomyopathy, and neuropathy. Moreover, ferroptosis inhibitors hold promise as potential pharmacological targets for mitigating diabetes-related complications. A better understanding of the role of ferroptosis in diabetes may lead to an improvement in global diabetes management.

In this review, we delve into the intricate relationship between ferroptosis and diabetes development, exploring associated complications and current pharmacological treatments.

## 1 Introduction

Diabetes mellitus (DM) is defined as a group of metabolic disorders characterized by chronic hyperglycemia resulting from deficiencies in insulin secretion, insulin action, or both. Globally, the prevalence of diabetes has surged to epidemic levels. Presently, it is estimated that over half a billion individuals are affected by diabetes worldwide, reflecting a global age-standardized diabetes prevalence of 6.1%. By 2050, projections indicate that this prevalence will more than double, with an estimated 1.31 billion individuals living with diabetes. Consequently, the prevention and management of diabetes remains a major challenge (1).

Ferroptosis is a specific and emerging form of cell death linked with various conditions including cancer, neurodegenerative diseases, cardiovascular diseases, diabetes, infections, inflammatory bowel disease, chronic lung disease, and acute kidney disease. Biochemically, ferroptosis is characterized by the accumulation of iron, increased production of lethal lipid reactive oxygen species (ROS), excessive lipid peroxidation, and depletion of the lipid repair enzyme glutathione peroxidase 4 (GPx4). It is considered a non-apoptotic regulated cell death mechanism, as it does not necessitate the involvement of caspases — a family of aspartate-specific cysteine proteases critical for the initiation and execution of apoptosis through the cleavage of specific intracellular substrates (**Table 1**). Ferroptosis is triggered by an iron-dependent accumulation of ROS and subsequent peroxidation of membrane polyunsaturated fatty acid phospholipids, leading to extensive oxidative damage. Morphologically, ferroptosis is characterized by mitochondria exhibiting shrinkage, high membrane density, reduced or vanished cristae, and a ruptured outer mitochondrial membrane. The major distinction between autophagy and ferroptosis lies in the absence of the formation of a classical closed bilayer membrane structure in the latter (2).

**Table 1:** Table: Characteristics of Different Types of Cell-Death.

Feature	Biochemical	Morphological	Genetic	Pathway Activation	References
**Ferroptosis**	Iron-dependent lipid peroxidation, Glutathione depletion, GPX4 inhibition	Mitochondrial shrinkage, cristae condensation, Membrane rupture	Upregulation of ACSL4, SLC7A11, and TFR1, Downregulation of GPX4	Iron metabolism pathways, ROS generation	(14, 88, 89)
**Apoptosis**	Caspase activation, DNA fragmentation, Cytochrome c release	Cell shrinkage, chromatin condensation, Membrane blebbing, apoptotic bodies formation	Upregulation of pro-apoptotic genes (e.g., BAX), Downregulation of anti-apoptotic genes (e.g., BCL-2)	Intrinsic (mitochondrial) and extrinsic (death receptor) pathways	(90–92)
**Autophagy**	Autophagosome formation, LC3 conversion, mTOR inhibition	Double-membrane autophagosomes, Cytoplasmic vacuolization	Upregulation of ATG genes, Downregulation of mTOR	AMPK and ULK1 pathways	(93–95)
**Necroptosis**	RIPK1, RIPK3, and MLKL activation, TNF-α signaling, ROS generation	Cell swelling, plasma membrane rupture, No formation of apoptotic bodies	Upregulation of RIPK1, RIPK3, MLKL phosphorylation	TNF-α, RIPK1/RIPK3 complex	(96–98)
**Pyroptosis**	Caspase-1/4/5/11 activation, GSDMD cleavage, IL-1β and IL-18 release	Cell swelling, pore formation in plasma membrane, Cellular lysis	Upregulation of NLRP3, IL-1β, and GSDMD, Caspase-1/11 dependent pathways	Inflammasome activation	(99–101)

Understanding the interplay between diabetes and ferroptosis holds potential for the development of novel pharmacological strategies.

In this review, we delve into the connection between ferroptosis and diabetes pathogenesis, associated complications, and potential treatments.

## 2 Mechanisms of ferroptosis and historical perspectives

**Figure 1:**
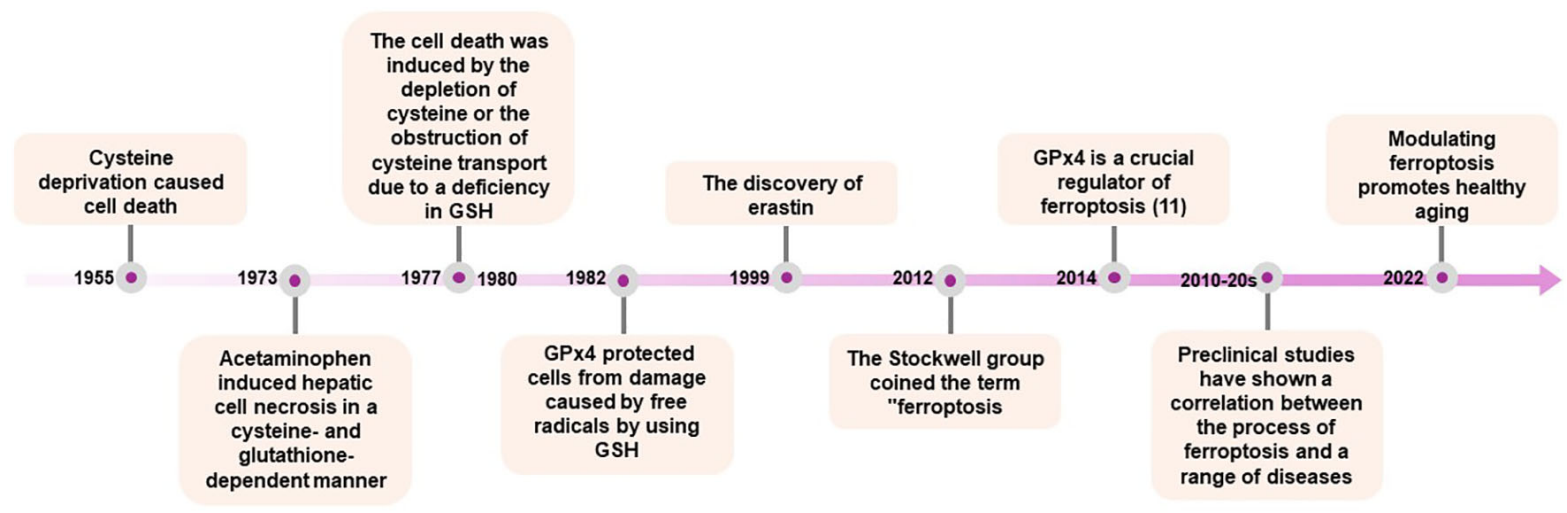
“Timeline of Key Discoveries in Ferroptosis Research”. This timeline outlines significant milestones in the study of ferroptosis, starting from the initial discoveries to the latest research advancements. It includes the identification of ferroptosis, the roles of GPx4 and lipid peroxidation, and the impact of ferroptosis on various diseases. Notable years and events such as the coining of the term “ferroptosis” by the Stockwell group, and the progression of research into its implications in health and disease are marked.

The groundwork in ferroptosis research traces back to the 1950s, initiated by Harry Eagles, who demonstrated that the deprivation of the amino acid cysteine induced cell death ​​in HeLa cells and mouse fibroblast strain L (3). In 1973, Mitchell identified that acetaminophen induced hepatic cell necrosis in rats was dependent on cysteine and glutathione (GSH) (**Figure 1**) (4). A few years later, Bannai showed that cysteine depletion in the medium or the inhibition of cystine transport let to reversible cell death mediated by GSH depletion, which could be prevented by vitamin E (5, 6). Concurrently, in 1982, GPx4 was identified as a selenoprotein capable of safeguarding against membrane lipid peroxidation in a GSH-dependent peroxidase manner (7). GPx4 belongs to the glutathione peroxidase enzyme family, consisting of a total of 8 members *(Gpx1-8)*, all capable of degrading fatty acid peroxide, alkyl peroxide, and hydrogen peroxide. However, Gpx4 remains the only enzyme with the capacity to degrade complex lipids and lipoproteins derived from cholesterol and phospholipids (8).

A significant milestone in the identification of ferroptosis occurred in 2003 with the discovery, through high-throughput screening, of a small molecule named erastin. Erastin was found to selectively induce non-apoptotic cellular death in an iron-dependent manner, specifically targeting oncogenic RAS (9). This form of cell death was found the be distinct from other types of cell death, such as apoptosis, autophagy, necroptosis, or pyroptosis, in terms of biochemical, morphological, and genetic characteristics. In 2012, the term ‘ferroptosis’ was coined to define this specific form of cell death, characterized by iron-dependent accumulation of reactive oxygen species. The Stockwell group described a novel non-apoptotic form of cell death induced by the inhibition of the amino acid antiporter system X_C_^−^, which facilitates the cellular uptake of cysteine and glutamate. This system is essential for providing cysteine, the precursor for GSH biosynthesis (10).

Two years later, the same research team published a study demonstrating that GPx4 is an essential regulator of this pathway (11). Preclinical investigations further confirmed the critical role of GPx4, as inducible Gpx4(-/-) mice generated in subsequent studies succumbed to acute kidney failure within two weeks of Gpx4 loss (12). These findings underscore the importance of ferroptosis in both physiological and pathological conditions, highlighting the need for a thorough understanding of its precise mechanisms and implications (13). Following these groundbreaking discoveries, numerous studies underscored the potential of ferroptosis and its modulation across various disease models, including neurodegenerative diseases, cardiovascular diseases, metabolic disorders, aging and oncology (14–16). These advances have enhanced our understanding of the complex molecular regulation of ferroptosis, which is discussed below.

## 3 Regulation of ferroptosis

**Figure 2:**
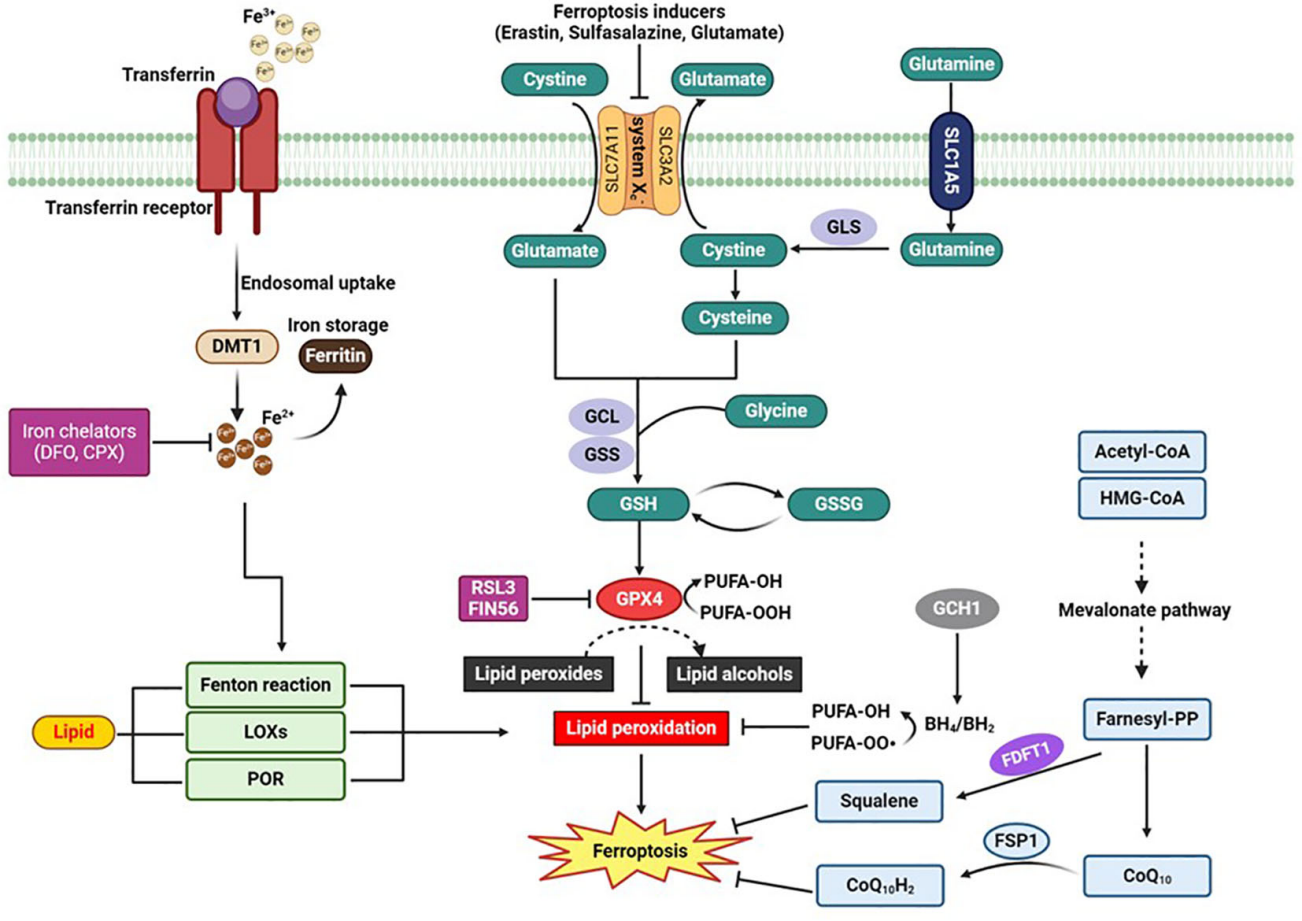
“Schematic Diagram of the Signaling Pathway of Ferroptosis”. This figure illustrates the key molecular mechanisms and pathways involved in ferroptosis, a regulated form of cell death dependent on iron and characterized by the accumulation of lipid peroxides. Key elements include the cystine/glutamate antiporter (system Xc-), glutathione (GSH), glutathione peroxidase 4 (GPx4), and the roles of iron chelators such as Deferoxamine (DFO) and Ciclopirox (CPX) in inhibiting ferroptosis. The diagram highlights how the disruption of these pathways can lead to the execution of ferroptosis. BH_2:_, dihydrobiopterin; BH_4_, tetrahydrobiopterin; CoQ_10_, coenzyme Q10; CoQ_10_H_2_, ubiquinol; CPX, Ciclopirox; CPX; DMT1, divalent metal transporter 1; DFO, Deferoxamine: FDFT1, farnesyl-diphosphate farnesyltransferase; FSP1, Ferroptosis suppressor protein 1; GCH1, GTP cyclohydrolase-1; GCL, glutamate-cysteine ligase; GLS, glutaminases; GPX4, glutathione peroxidase 4; GSH, glutathione; GSS, glutathione synthetase; GSSG, oxidized glutathione; LOXs, lipoxygenases; POR, cytochrome p450 oxidoreductase; RSL3, RAS-selective lethal 3.

There are several distinct cellular mechanisms that regulate the development of ferroptosis. GPx4 is widely recognized as a master regulator of ferroptosis, however, both GPx4 dependent and independent pathways are associated with this process. GPx4, which is present in both the cytosol and mitochondria, serves as the primary defense against lipid peroxidation by catalyzing the reduction of toxic lipid peroxides to non-toxic alcohols (17). Pharmacological or genetic inhibition of GPx4 is associated with rapid lipid peroxidation and the characteristic features of ferroptosis (11) (**Figure 2**). Acyl—CoA synthetase long-chain familymember4 (ACSL4) also play a crucial role in promoting ferroptosis by regulating the metabolism of arachidonic acid, eicosapentaenoic acid and lipid peroxidation that is linked to GPx4 (18).

Recently accumulating evidence indicated the existence of GPX4-independent pathways in the suppression of ferroptosis (13, 19). Ferroptosis suppressor protein 1 (FSP1), also known as AIFM2, has emerged as a significant player in ferroptosis suppression independent of GPX4 (20, 21). FSP1 regenerates reduced CoQ10 using NADPH, a critical source of cellular reducing power. Reduced CoQ10 scavenges lipid peroxidation intermediates, thus preventing ferroptosis (**Figure 2**). The role of NADPH in regenerating CoQ10 has been highlighted as a biomarker for ferroptosis resistance in cancer cell lines, with the cytosolic phosphatase MESH1 influencing ferroptosis sensitivity by modulating NADPH levels (22, 23). Another pivotal mechanism involves GTP cyclohydrolase 1 (GCH1), which produces tetrahydrobiopterin (BH_4_). BH_4_ acts as a lipophilic antioxidant similar to CoQ10, preventing lipid peroxidation and remodeling lipid membranes to increase reduced CoQ10 while decreasing PUFA-PLs (**Figure 2**) (24, 25). CRISPR screens have identified GCH1 as a critical regulator of ferroptosis sensitivity (25). Additionally, dihydroorotate dehydrogenase (DHODH) functions within mitochondria to reduce CoQ10, offering another layer of protection against ferroptosis (26). Cells with high DHODH expression are more resistant to ferroptosis, while those with low expression are more sensitive. Further expanding the landscape of GPX4-independent ferroptosis suppression, interleukin-4-induced-1 (IL4i1) generates indole-3-pyruvate (In3Py), which not only scavenges radicals but also modulates gene expression to reduce lipid peroxidation (27). This pathway suggests the potential existence of other endogenous metabolites that can suppress ferroptosis by interfering with radical intermediates or gene regulation. The identification of these diverse mechanisms underscores the complexity of ferroptosis regulation and highlights multiple therapeutic targets for diseases where ferroptosis plays a crucial role. Each mechanism provides unique insights into cellular defense strategies against lipid peroxidation, opening new avenues for research and therapeutic development.

## 4 Iron metabolism and ferroptosis

Iron is an important trace element involved in various cellular processes, such as erythropoiesis, oxygen transport, and energy metabolism. The primary source of iron is dietary, with absorption occurring in the intestines in the form of heme iron or free Fe^2+^. Approximately 60% of iron is bond to erythrocytes, while a quarter is stored in ferritin and hemosiderin within bone marrow, spleen, and liver. Another 5% in found in myoglobin, with less than 1% transported by transferrin.

Iron homeostasis is finely regulated, balancing intracellular utilization, storage, and uptake processes. Physiologically, ferritin sequesters and stores iron as a protective measure against iron-induced oxidative stress. Circulating iron exists predominantly as Fe^3+^, which is imported into cells by transferrin. The transferrin receptor (TfR1) mediates the endocytosis of iron and transferrin within clathrin-coated vesicles. Following endocytosis, Fe^3+^ is reduced to Fe^2+^ within the lysosomes. Subsequently, the zinc iron regulatory protein family 8/14 (ZIP8/14) and the divalent metal transporter 1 (DMT1) release the oxidized form of iron into the labile iron pool. Excessive levels of Fe^2+^ promote the production of ROS through the Fenton reaction, amplifying lipid peroxidation and consequently triggering the initiation of ferroptosis. The induction of ferroptosis then appears as a factor favoring the pathogenesis of diabetes via several actions that are discussed below.

## 5 Role of ferroptosis and iron metabolism in pancreatic β-cells dysfunction

Iron deficiency or excess can affect glucose metabolism, and conversely, hyperglycemia can lead to iron overload. This close relationship between iron and glycemia is illustrated by the association between elevated ferritin levels and the development of type 2 diabetes (28–30). The deleterious effects of iron overload were initially identified in pathological conditions of excess iron, such as hereditary hemochromatosis, characterized by the presence of several elements including diabetes, hepatic steatosis, and cardiomyopathy. The disruption of glucose homeostasis stems from a defect in insulin secretion caused by pancreatic β-cells dysfunction induced by iron overload, which can be mitigated by phlebotomy or iron chelation.

Iron metabolism and ferroptosis both play roles in glucose homeostasis, affecting both insulin secretion and resistance. At the level of the β-cells of the pancreas, iron is involved in insulin secretion. It is incorporated into β-cells of the pancreas by the mechanism discussed in section 4 (31). Inside the cell, iron participates in the mechanism of insulin secretion by promoting ROS production through the Fenton reaction, which is considered an enhancing signal for insulin secretion (32). Iron overload can lead to pancreatic β-cells failure and apoptosis through several mechanisms, such as ROS generation, reduced capacity of detoxification enzymes, or the enhancement of amylin ß-sheet formation, leading to aggregate deposition (33). Furthermore, iron serves as a cofactor for various enzymes and plays a significant role in Fe-S cluster formation, impacting β-cell proliferation, differentiation, and insulin secretion (34). Pancreatic β-cells from iron-deficient mice exhibit reduced glucose-induced insulin secretion (GSIS) capacity (35). Taken together, these findings suggest that both iron depletion and overload adversely affect pancreatic β-cells. Therefore, iron levels should be carefully regulated to avoid excess or deficiency, in order to preserve the secretory function of islet β-cells.

Evidence suggests that ferroptosis is implicated in pancreatic β-cell function and survival. These cells have low expression levels of several antioxidant enzymes, such as catalase, GSH peroxidase, and superoxide dismutase, which may predispose them to the accumulation of ROS and consequently, the induction of ferroptosis (36). On one hand, *in vitro* studies have shown that erastin, a ferroptosis inducer, reduces GSIS. On the other hand, iron-1, a ferroptosis inhibitor, exhibits protective effects on GSIS ability (37). Pancreatic β-cell death is a critical factor in the development and progression of diabetes, and ferroptosis appears to play a role in this process. In pancreatic β-cells, the induction of ferroptosis is linked to significantly accelerated cell death. Conversely, inhibiting ferroptosis with Ferrostatin-1 has been shown to enhance the survival of these cells (37). A recent study demonstrated that in pancreatic islets, RSL3 induces oxidative stress, leading to an increase in intracellular iron and elevated expression of ACSL4 protein, which in turn results in a significant reduction in islet function (38). Collectively, these findings suggest that inhibiting ferroptosis may protect pancreatic β-cell function and survival.

## 6 Insulin resistance

In addition to their involvement in β-cell function and survival, iron and ferroptosis are also implicated in insulin resistance (IR), a multifaceted pathophysiological state characterized by a reduced response of insulin-depend cells such as hepatocytes, skeletal muscle cells, or adipocytes. In the liver, iron accumulation in pathological states such as hemochromatosis has been shown to trigger gluconeogenesis, impacting glucose levels (39, 40). Excess iron in hepatocytes induces the generation of ROS via the Fenton reaction, similar to observations in other cell types. This leads to the activation of several enzymes such as NADPH oxidases (NOXs) and arachidonate lipoxygenases (ALOXs), triggering lipid peroxidation, cellular membrane destruction, and ultimately, ferroptosis (41).

In preclinical models of non-alcoholic steatohepatitis (NASH), ferroptosis is associated with the development of hepatic inflammation, while inhibition of ferroptosis may protect against NASH progression (42, 43). Ferroptosis exacerbates hepatic steatosis and IR by triggering the unfolded protein response, which in turn stimulates hepatic lipogenesis. This sets off a vicious cycle involving iron accumulation, ferroptosis, hepatic lipid accumulation, and IR, ultimately impairing glucose homeostasis (44).

Adipocytes also play a crucial role in the association between ferroptosis and diabetes through several mechanism. Firstly, the inflammatory state observed in adipose tissue in diabetes may inhibit the NRF2-GPX4 pathway, leading to ferroptosis in the vagus nerve. This reduces the nerve’s capacity to transmit signals detected and collected by sensory nerves to the central nervous system, causing autonomic imbalance and disrupting adipose tissue homeostasis. Secondly, regarding the immunological aspect of adipose tissue, the accumulation of iron often observed in diabetes can activate ferroptosis in M2 macrophages, T regulatory cells, and B lymphocytes by reducing the levels of NRF2 and GPx4. Further research is needed to elucidate the precise mechanisms linking ferroptosis in adipose tissue and diabetes (45).

Skeletal muscle cells play a pivotal role in insulin sensitivity/resistance, as they are responsible for approximately 80% of total body insulin-stimulated glucose uptake. Excess iron has been shown to induce insulin resistance, leading to disruption in glucose homeostasis. However, the role of ferroptosis in skeletal muscle tissue remains less studied thus far (46). The control and modulation of ferroptosis may therefore represent a potential avenue for maintaining glucose homeostasis.

## 7 Ferroptosis and diabetes treatment

Several pharmacological treatments for diabetes may modulate ferroptosis. Metformin, a synthetic biguanide and the first line-therapy for type 2 diabetes, is the most widely used pharmacological treatment. Its mechanisms of action are complex. Metformin appears to act by activating the adenosine monophosphate-activated protein kinase (AMPK) signaling pathway, as well as other AMPK-independent pathways. These actions suppress hepatic gluconeogenesis and enhance insulin-stimulated peripheral glucose uptake, altogether leading to improved glucose regulation in diabetic individuals (47). Some *in vitro* and preclinical studies suggest that metformin modulates ferroptosis. In several breast cancer cell lines, metformin induces ferroptosis via different mechanisms, such as inhibiting UFMylation of SLC7A11 and targeting the miR-324-3p/GPX4 axis, suggesting another potential anti-cancer property of metformin (48, 49). In an *in vivo* study with a rat-model of vascular calcification, metformin attenuated vascular smooth muscle cell (VSMC) calcification through anti-ferroptosis effects (50). Among the potential molecular mechanisms by which metformin influences ferroptosis, a reduction in iron overload in the liver has been demonstrated in a preclinical model of non-alcoholic fatty liver disease (NAFLD) via the AMPK-ferroportin pathway (51). Another identified mechanism involves metformin’s modulation of the gut microbiota, which is characterized by an increase in gamma-aminobutyric acid (GABA)-producing bacteria, leading to the inhibition of ferroptosis (52)..

Sodium-glucose cotransporter 2 (SGLT2) inhibitors are a pharmacological therapeutic class that decreases glycemia by blocking glucose reabsorption in the proximal tubules, leading to glucosuria. This therapeutic class has not only shown improvements in blood sugar levels but also cardiac and renal protection benefits in both diabetics and non-diabetics, leading to an increase in its use. SGLT2 inhibitors may reduce ferroptosis and promote various beneficial effects observed in preclinical studies. These effects include enhancing the revascularization of ischemic hindlimbs in diabetic mice, improving cardiac function in a rat model of heart failure with preserved ejection fraction, and promoting tubular kidney function (53–55). Overall, SGLT2 inhibitors promote cardiometabolic health, at least partly through a reduction of ferroptosis. Mechanistically, this reduction is mediated through the induction of sirtuin-1 and an increase in intracellular levels of glutathione, both of which enhance glutathione-dependent glutathione peroxidase 4 (56).

Glucagon-like peptide-1 receptor agonists (GLP-1 RA) are widely used treatments for individuals with type 2 diabetes, known to improve glucose homeostasis and promote weight loss in overweight or obese patients. Preclinical studies suggest a potential reduction of ferroptosis by liraglutide, with a decrease in hepatic iron accumulation observed in db/db mice, thereby reducing insulin resistance and the development of hepatic steatosis (57). Additionally, another study showed a reduction in iron deposition in the hippocampus, leading to reduced damage in hippocampal neurons and improvement in synaptic plasticity, thereby favoring cognitive function restoration in db/db mice (58).

Dipeptidyl-peptidase-4 (DPP-4) inhibitors act on glucose levels by blocking the degradation of GLP-1 and glucose-dependent insulinotropic peptide (GIP). Interestingly, the activity of the DPP-4 enzyme appears to be involved in ferroptosis due to its interaction with the tumor suppressor P53. In human colorectal cancer cells (CRC), the loss of P53 counteracts the deposition of nuclear DPP4 and instead promotes the formation of a complex between DPP-4 and NADPH oxidase 1 (NOX1) at the plasma membrane, thus promoting lipid peroxidation. Conversely, P53 inhibits ferroptosis by decreasing DPP-4 activity. Furthermore, several DPP4 inhibitor molecules completely inhibit erastin-induced ferroptosis in CRC cells, suggesting a bidirectional relationship between DPP-4 and P53 in the context of ferroptosis (59).

## 8 Ferroptosis in diabetes-related complications

**Figure 3:**
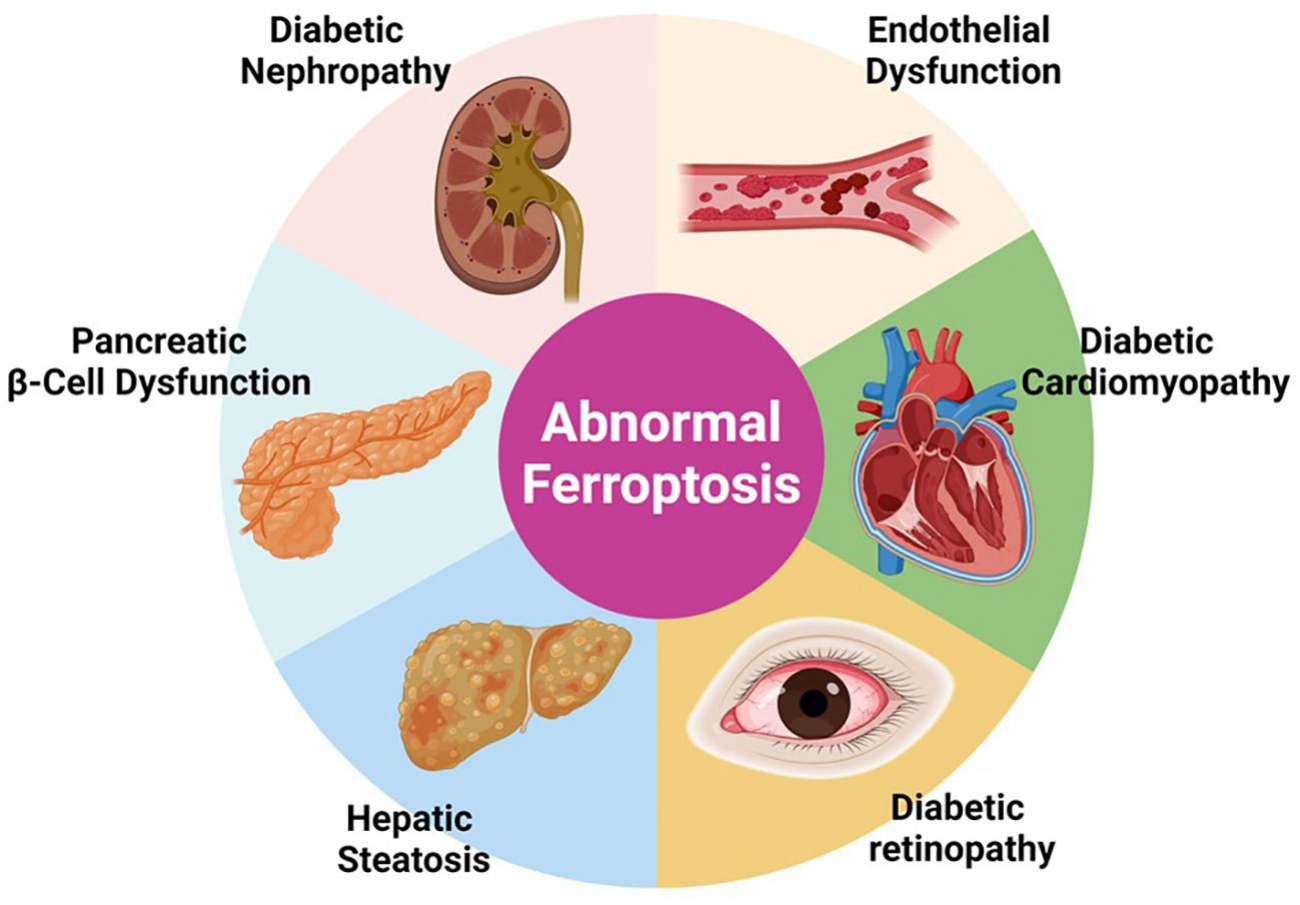
“Implications of Ferroptosis in Diabetic Complications”. This figure presents the involvement of abnormal ferroptosis in various diabetic complications including diabetic nephropathy, endothelial dysfunction, pancreatic β-cell dysfunction, diabetic cardiomyopathy, and hepatic steatosis. The diagram shows how dysregulated ferroptosis contributes to the pathophysiology of these conditions, emphasizing the potential of targeting ferroptosis pathways for therapeutic interventions in diabetes-related diseases.

DM is associated with several complications that are traditionally categorized into macro- and microvascular based on the diameter of the vessels of the affected organs. Macrovascular complications involve diseases such as coronary heart disease (CAD), cerebrovascular disease, and peripheral arterial disease (PAD). Microvascular complications, on the other hand, encompass conditions like diabetic retinopathy (DR), diabetic kidney disease (DKD), and diabetic peripheral neuropathy (DPN) (**Figure 3**). Ferroptosis may contribute to the development of complications associated with diabetes due to shared cellular metabolic pathways and disturbances characteristic of this form of cell death. These include dysregulation of iron homeostasis, iron overload, reduced antioxidant capacity, ROS accumulation, and mitochondrial and endothelial dysfunction. Moreover, the hyperglycemic environment in diabetes further promotes the activation of pathways linked to ferroptosis. This suggests potential connections and reciprocal effects between diabetes, ferroptosis, and the associated complications.

### 8.1 Diabetic retinopathy

DR is characterized by damage to the retina, which can lead to vision impairment and affects approximately one-third of individuals with DM. Prolonged hyperglycemia in DM induces microvascular injury, promoting retinal hypoxia, neovascularization, reduced retinal permeability, microaneurysms, hemorrhages, and macular edema. The precise molecular and cellular mechanisms underlying the development of DR remain only partially understood but include oxidative stress with the accumulation of ROS, leading to mitochondrial dysfunction, inflammation, neuroglial dysfunction, endothelial progenitor cell dysfunction, and cellular apoptosis, ultimately resulting in progressive alterations in the function and the structure of the retina.

Chronic hyperglycemia reduces the proliferation of human retinal capillary endothelial cells (hRCE), a phenomenon that can be amplified by ferroptosis through the ubiquitination of GPX4, enhanced by TRIM46 (60). TRIM46 facilitates the induction of increasing the permeability and inflammation of hRCE by ferroptosis via the ubiquitination of IκBα (61). Furthermore, inhibition of the adipokine FABP4 has been shown to reduce lipid peroxidation in a mice model of DR through the modulation of ferroptosis, highlighting another aspect of the involvement of ferroptosis in DR. However, further research is needed to fully understand the role of ferroptosis in DR (62).

### 8.2 Diabetic kidney disease

Diabetic kidney disease (DKD) represents the leading cause of chronic kidney disease and end-stage kidney disease (ESKD), necessitating dialysis or renal transplantation. It is characterized by a progressive decline in glomerular filtration rate and/or proteinuria. The involvement of ferroptosis in DKD has been more widely studied, with more data available compared to DR. Several *in vitro*, preclinical animal, and human studies have shown evidence of the implication of ferroptosis in the development of DKD, including excess iron content or transferrin levels in tubular kidney cells, as well as increased expression levels of GPX4 and SLC7A11 (63, 64). The precise mechanisms linking the presence of diabetes to the development of the biological phenomenon of ferroptosis at the renal level remain to be elucidated. Nevertheless, it can be hypothesized that the hyperglycemic state and the production of ROS participate in the induction of ferroptosis.

Ferroptosis in the kidney appear to affect several cell types including mesangial cells, podocytes, or tubular cells. The increase in the expression of GPX4 and SLC7A11, along with the reduction of iron accumulation via upregulation of Prdx6, allows for a reduction in *in vitro* damage to podocytes in a model of DKD (65). High mobility group box 1 (HMGB1) is a molecule frequently found in cases of inflammatory damage, particularly in cases of DKD. Its inhibition reduces *in vitro* ferroptosis in mesangial cells induced by a medium high in glucose, as evidenced by a decrease in ACSL4 and an increase in GPX4 (66). Podocytes, a cell type playing a major role in glomerular filtration barrier integrity, has been shown to display ferroptosis activation upon high glucose exposure through the antioxidant molecule peroxiredoxin 6 (Prdx6) (65). Ferroptosis is also implicated in the process of renal tubular cell death, as evidenced by a reduction in GPx4 levels, accumulation of lipid peroxidation, and an increase in the expression level of acyl-CoA synthetase in both *in vitro* and *in vivo* preclinical models of renal tubular epithelial cell apoptosis (63).

Several hypotheses currently link diabetic kidney disease (DKD) to ferroptosis. For instance, renal ischemia, commonly observed in diabetes, induces the overexpression of hypoxia-inducible factor (HIF), particularly heme oxygenase-1 (HO1). This leads to the mobilization of the labile iron pool in the kidneys, potentially promoting ferroptosis (67, 68). Another proposed mechanism involves the hyperglycemic state, which inhibits nuclear factor E2-related factor 2 (Nrf2), a regulator with antioxidant and anti-inflammatory properties, thereby inducing ferroptosis in the kidneys (66). Additionally, recent research has shown that hydrogen sulfide (H2S) or sulfide metabolism, which is being explored as a promising approach for renal protection, can inhibit renal ferroptosis and the progression of DKD by reducing basement membrane thickening, mesangial expansion, and renal fibrosis (69, 70).

All of this indicates an involvement of ferroptosis in various mechanisms and cell types, suggesting that it could represent a new potential avenue for therapeutic approaches to DKD.

### 8.3 Diabetic cardiomyopathy

Diabetic cardiomyopathy (DCM) is defined by adverse myocardial structural remodeling and altered function in the absence of classical etiologies such as coronary artery disease, valvopathy, or hypertension. The pathogenesis of DCM is the subject of extensive research; it appears complex, involving numerous pathways, and remains only very partially elucidated. Among the mechanisms contributing to DCM are mitochondrial dysfunction, excessive production of ROS, cardiomyocyte death, endoplasmic reticulum stress, endothelial damage, and cardiac fibrosis. There is growing evidence indicating a role for ferroptosis in the pathogenesis of DCM. For instance, the accumulation of lipids and ROS within cardiomyocytes exacerbates the development of DCM. Additionally, the importance of iron metabolism in myocardial function and its accumulation in DCM suggest a role for ferroptosis in this condition. Furthermore, mitochondrial dysfunction, which is also present in DCM, can promote ferroptosis (71).

Firstly, in a preclinical model of type 2 diabetes, a high-fat, high-sucrose diet was shown to cause hypertrophy, lipid peroxidation, and mitochondrial dysfunction in the heart (72). Further evidence for the involvement of ferroptosis comes from the fact that overexpression of mitochondrial phospholipid hydroperoxide glutathione peroxidase 4 (mPHGPx) provides cardiac protection in a model of DCM induced by streptozotocin (73). Additionally, several studies have demonstrated the role of NRF2, a major actor in the cellular antioxidant response, particularly by acting as transcriptional regulator of anti-ferroptotic genes in DCM (74, 75). Indeed, the inhibition of NRF2 via autophagy in a model of cardiomyocyte-restricted (CR) knockout of the autophagy-related 5 gene (CR-Atg5KO) accelerates the progression of DCM in mice (76). Conversely, the induction of NRF2 levels by sulforaphane increases metallothionein, leading to protection against the development of DCM (77, 78). On the other hand, NRF2 activators such as 6-gingerol and curcumin have shown a protective effect *in vitro* and *in vivo* (79, 80). Inhibition of ferroptosis via activation of NRF2 could therefore represent a new therapeutic avenue in the treatment of DCM.

### 8.4 Endothelial dysfunction

Diabetes is a major risk factor in the development of atherosclerosis and associated diseases such as coronary heart disease, ischemic stroke, or peripheral arterial disease. Chronic hyperglycemia, acute glucose fluctuations, and IR elicit oxidative stress, inducing endothelial dysfunction (ED). This phenomenon is characterized by reduced nitric oxide (NO) bioavailability, vasoconstriction, and a pro-inflammatory and pro-thrombotic state. ED serves as a major hallmark and a poor prognostic marker for micro- and macrovascular complications associated with diabetes. Several markers of ferroptosis activation in vascular smooth muscle cells (VSMCs), vascular endothelial cells (VECs), and macrophages are positively correlated with the development of atherosclerotic plaques (81). Ferroptosis induction may aggravate the atherogenic process, while anti-ferroptosis molecules such as Fer-1 may inhibit atherosclerosis development. Therefore, inhibiting ferroptosis may represent a potential therapeutic target to prevent the development of ischemic diseases, particularly among diabetic individuals.

### 8.5 Ferroptosis modulation to target diabetes

While preclinical data on the impact of modulating ferroptosis in diabetes and its complications are promising, available human clinical data remain limited. Among the candidate molecules, Bardoxolone methyl (BXM), currently in phase 3 clinical trials for the treatment of diabetic kidney disease (DKD), inhibits ferroptosis by promoting Nrf2 activation. Rosiglitazone, a peroxisome proliferator-activated receptor-gamma (PPAR-γ) agonist, also exhibits ferroptosis-inhibitory activity by blocking ACSL4, thereby reducing iron overload and lipid peroxidation (82). Although Rosiglitazone has shown positive effects in diabetes, particularly by increasing insulin sensitivity, it is potentially associated with an increased risk of cardiovascular problems and certain cancers ( (83). Quercetin, a plant flavonoid, also possesses anti-ferroptotic properties and has demonstrated beneficial effects on blood glucose control and diabetes complications in some clinical trials. However, large-scale and long-term studies are necessary to confirm the efficacy of this compound in diabetes treatment (84). Curcumin, a polyphenol found in *Curcuma longa*, has demonstrated anti-inflammatory, antioxidant, and ferroptosis-blocking properties. It has shown potential in improving various metabolic parameters, including glucose homeostasis, in some clinical trials, and may offer protection against the progression of diabetes complications such as DKD (85, 86).

A number of compounds targeting ferroptosis more specifically are currently under development and evaluation, primarily at the preclinical stage. These include GPx4 activators or inhibitors such as ML162, ML210, and compound 1d4, as well as FSP1 activators like NPD4928 and iFSP1 (87). Overall, more clinical data are needed to assess the potential of targeting ferroptosis as a therapeutic strategy for diabetes.

## 9 Conclusion

Ferroptosis, a specific type of cell death characterized by a lethal accumulation of ROS and peroxidation of membrane lipids in an iron-dependent manner, remains only partially understood regarding its mechanistic underpinnings and its involvement in various medical conditions such as diabetes.

Ferroptosis and iron metabolism seem to play significant roles in multiple aspects of diabetes pathophysiology, both in terms of beta cell dysfunction and IR.

This review aims to consolidate the existing knowledge on the involvement of ferroptosis in diabetes pathogenesis, its associated complications, and the impact of anti-diabetic treatments on ferroptosis.

Ongoing efforts are directed towards a comprehensive understanding of ferroptosis, presenting promising therapeutic avenues not only in diabetes but also in other fields like oncology.
